# A Cyclic-di-AMP Adjuvanted CPAF Protein Vaccine Is Immunogenic in Swine, but It Fails to Reduce Genital *Chlamydia trachomatis* Burden

**DOI:** 10.3390/vaccines13050468

**Published:** 2025-04-27

**Authors:** Leonie Bettin, Maria Stadler, Christine Unterweger, Maximiliane Dippel, Jonathan M. Harris, Andrea Buzanich-Ladinig, Taylor B. Poston, Toni Darville, Tobias Käser

**Affiliations:** 1 Department of Biological Sciences and Pathobiology, Center of Pathobiology, Immunology, University of Veterinary Medicine Vienna, 1210 Vienna, Austria; leonie.bettin@vetmeduni.ac.at (L.B.);; 2 Clinical Department for Farm Animals and Food System Science, Clinical Centre for Population Medicine in Fish, Pig and Poultry, University of Veterinary Medicine Vienna, 1210 Vienna, Austria; christine.unterweger@vetmeduni.ac.at (C.U.); andrea.ladinig@vetmeduni.ac.at (A.B.-L.); 3 Centre for Immunology and Infection Control, Queensland University of Technology, Brisbane, QLD 4000, Australia; j2.harris@qut.edu.au; 4 Department of Pediatrics, University of North Carolina at Chapel Hill, Chapel Hill, NC 27599, USAlad@email.unc.edu (T.D.)

**Keywords:** *Chlamydia trachomatis*, vaccination, adjuvant, chlamydial-protease-like activity factor, animal model, pigs, STING agonist, c-di-AMP

## Abstract

**Background/Objectives**: *Chlamydia trachomatis* (*Ct*) is the leading bacterial cause of sexually transmitted infection globally. If undiagnosed or left untreated, these infections can lead to serious complications such as infertility, ectopic pregnancies, and chronic pelvic pain. Despite the high prevalence and potential for serious health complications, no vaccine has been licensed. Pigs offer a valuable biomedical model for chlamydia research: they have an overall high degree of similarity to humans and serve as natural hosts for *Chlamydia suis* (*Cs*), a close relative of *Ct*. Thus, in this study, the pig model was used to evaluate a vaccine candidate against *Ct*. **Methods**: The vaccine candidate consists of chlamydial-protease-like activity factor (CPAF) protein adjuvanted with STING (Stimulator of Interferon Genes) pathway agonist cyclic-di-AMP (c-di-AMP). Pigs received two doses intramuscularly followed by two intranasal doses. Each week, the systemic T cell response was assessed via IFN-γ and IL-17 ELISpots, as well as multi-parameter flow cytometry on 0, 14, and 28 days post vaccination (dpv). The humoral immune response was analyzed by measuring CPAF-specific antibody levels and avidity via ELISAs. **Results**: Vaccination with c-di-AMP adjuvanted CPAF triggered low-level systemic IFN-γ and multifunctional IFN-γ^+^TNF-α^+^ CD4 T cell responses. Despite the rather low systemic effector cytokine production, robust anti-CPAF IgG responses were detected in serum, vaginal swab eluates, and oviduct flushes. Genital *Ct* challenge 42 dpv resulted in only transient infection, precluding a confident assessment of vaccine efficacy of the tested CPAF/c-di-AMP vaccine candidate. However, after challenge, vaccinated pigs exhibited boosted systemic anti-CPAF IFN-γ and mucosal IgG responses compared to unvaccinated pigs. **Conclusions**: Thus, while vaccine efficacy remains elusive, the CPAF/c-di-AMP vaccine candidate was immunogenic: it elicited a low-level systemic cell-mediated response and robust humoral immune responses. Future studies will incorporate a STING agonist directly conjugated to CPAF as well as addition of other Th1-inducing adjuvants to enhance cellular immunity.

## 1. Introduction

*Chlamydia trachomatis* (*Ct*) is an obligate intracellular bacterium with a biphasic developmental cycle that includes two distinct forms, infectious elementary bodies and dividing reticulate bodies (reviewed in [1]). According to the WHO, *Ct* is one of the major causes of sexually transmitted infections (STIs) with an estimated 128.5 million new cases globally in 2020. Since *Ct* infections are often asymptomatic, the bacteria can remain undetected and lead to serious complications in women such as pelvic inflammatory disease (PID). Repeated *Ct* infections have been linked to an increased PID risk [2]. PID results from ascending spread of *Ct* to the upper reproductive tract and is characterized by a sustained inflammatory microenvironment leading to tubal pathology with chronic pain, ectopic pregnancies, and tubal infertility (reviewed in [3]). The steady rise in reported *Ct* infections annually and the risk of serious complications highlight the need for effective prevention, with vaccines offering a promising solution.

Currently, multiple *Ct* vaccine candidates are being evaluated in pre-clinical testing (reviewed in [4]), and three candidates have been evaluated in phase I clinical trials thus far: CTH522, consisting of peptomers of the chlamydial major outer membrane protein (MOMP), adjuvanted with CAF01 liposomes or aluminum hydroxide [5], and CTH522 adjuvanted with CAF09b including an investigation of the ophthalmic immunization route [6]. However, no vaccine is publicly available yet, and continuous effort in pre-clinical testing with suitable animal models is needed. While mice are commonly used, their inbred nature and physiological differences limit their translation to humans. Pigs, however, share key anatomical, physiological, and immunological similarities with humans [7,8,9]. These characteristics have led to their use in biomedical research, including *Ct* vaccine development [10,11,12,13]. Moreover, pigs are the natural host for *Chlamydia suis* (*Cs*), a close phylogenetic relative of *Ct* and linked to similar pathologies like conjunctivitis, pneumonia, enteritis, and reproductive disorders [14,15,16]. Since *Cs* infection is ubiquitous among pigs, treatment of pregnant sows or delivery by cesarean section followed by separation of piglets from their mothers is required if *Cs*-naïve pigs are to be examined. Previously, we have shown that both *Cs* and *Ct* can infect *Cs*-seropositive pigs, and that CD4 T cell responses are cross-reactive [17,18]. This research established the basis for utilizing *Cs*-pre-exposed pigs as a relevant animal model for *Ct* vaccine development that mimics the *Ct* pre-exposed human population in clinical trials.

A protective immune response against *Ct* is dependent on CD4 T cell responses. In particular, CD4 T cells that secrete IFN-γ (Th1 cells) play a crucial role in pathogen clearance [19,20,21]. The role of antibodies is less clear and still debated. While anti-*Ct* antibodies can be detected in most women with *Ct* infection, they do not seem to be associated with protection [22,23,24]. In mice, anti-chlamydia antibodies are not essential for resolving a primary infection but may help reduce bacterial load during re-infections [25,26]. Hence, while antibodies seem to be beneficial for protection, developing a *Ct* vaccine that induces a strong Th1 response is of utmost importance. This directed induction of a Th1 response requires an immunogenic protein as well as a safe and effective adjuvant. We have previously demonstrated that a vaccination with UV-inactivated *Cs* in the pig model can induce robust IFN-γ secretion by CD4 T cells, which also resulted in a reduced genital *Cs* load upon challenge compared to unvaccinated controls, highlighting the suitability of the *Cs*-pre-exposed pig model [18]. Following the successful establishment of the *Cs*-pre-exposed outbred pig model, we evaluated the immunogenicity of a *Ct* vaccine candidate (TriAdj-adjuvanted chlamydial-protease-like activity factor (CPAF)) using various vaccination strategies and administration routes. By assessing the humoral and cell-mediated immune response, we were able to show that the vaccine candidate is highly immunogenic when administered twice intramuscularly (IM) followed by two intranasal (IN) doses [13].

We now used the same IM/IN vaccination regimen to assess the immunogenicity and efficacy of another *Ct* vaccine candidate, CPAF adjuvanted with the STING (Stimulator of Interferon Genes) agonist c-di-AMP. The vaccine antigen was originally chosen based on its immunoprevalence in a cohort of *Ct*-seropositive women. A study tested T cell recognition of various *Ct* proteins in *Ct*-infected female patients. In this patient cohort, CPAF was the most immunoprevalent antigen and elicited a positive T cell response in >50% of enrolled women. Further, epitope mapping revealed multiple CD4 T cell epitopes across the protein, making CPAF a very promising vaccine immunogen [27]. The serine protease CPAF is secreted by *Ct* into the host cell cytoplasm, where it seems to modulate varied host functions reviewed in [28]. For example, CPAF plays a role in blocking NF-κB p65 nuclear translocation, leading to reduced IFN-β production, regulating CXCL10 levels and preventing the activation of neutrophils [29,30,31]. In the current study, the vaccine antigen CPAF was adjuvanted with the bacterial second messenger molecule c-di-AMP. In mammals, c-di-AMP binds the receptor STING leading to the production of proinflammatory cytokines and type I interferons which seems to promote a Th1 bias in the subsequent adaptive immune response [32]. When compared to poly(I:C)/CpG in mice vaccinated with ovalbumin, c-di-AMP resulted in significantly higher antigen-specific IgG levels, CD8 cytotoxic responses, and Th1 and Th17 responses [33]. Similarly, Van Dis et al. observed that STING-activating cyclic dinucleotides (CDNs) formulated in a protein subunit vaccine against *Mycobacterium tuberculosis* stimulate CD4 T cells: they elicited strong antigen-specific Th1 and Th17 responses and protection against *M. tuberculosis* [34]. Moreover, c-di-AMP combined with CPAF has already shown promising results in mice against intravaginal challenge with *Chlamydia muridarum* (*Cm*). Not only did it elicit stronger memory T cell responses compared to the adjuvants CpG and AS03, but it also resulted in reduced cervical chlamydial burden upon challenge [35].

Hence, in this study, we tested c-di-AMP adjuvanted CPAF (CPAF/c-di-AMP) as a vaccine candidate in our *Cs*-pre-exposed pig model. Our aims were to determine both vaccine immunogenicity and efficacy in regard to lowering bacterial burden following a *Ct* challenge.

## 2. Materials and Methods

### 2.1. Animal Trial

**Figure 1:**
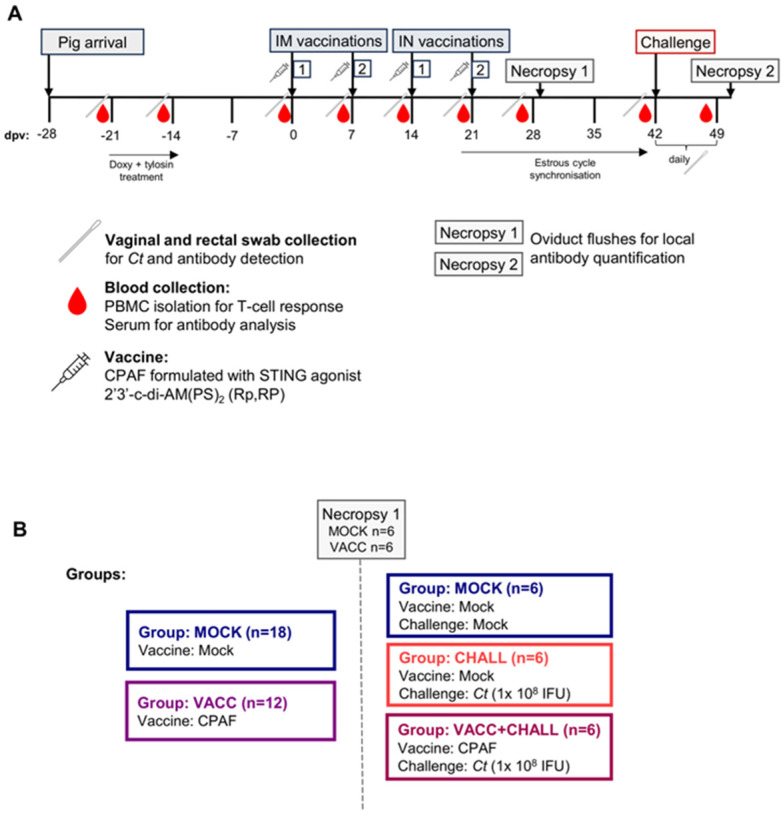
*Ct* CPAF vaccination trial layout and groups. (**A**) Upon arrival, the *Cs*-pre-exposed outbred pigs were treated with doxycycline and tylosin for 7 days to eliminate ongoing *Cs* infections, followed by a 2-week resting period to allow the anti-*Cs* response to decline. Pigs were then vaccinated at day 0 and 7 intramuscularly (IM) and at day 14 and 21 intranasally (IN). Throughout the trial, weekly blood and swab (vaginal and rectal) collections took place as outlined in the timeline. At 28 days post (first) vaccination (dpv), the pre-challenge necropsy took place with n = 6 MOCK and n = 6 VACC pigs, while remaining pigs were kept, and estrous cycles were synchronized to facilitate the post-cervical challenge at 42 dpv using MOCK or 1 × 10^8^ IFU of *Ct*. After a week of daily swab collections to monitor *Ct* load, the pigs were scarified to collect oviduct flushes for local antibody quantification. (**B**) shows the group assignments. A total of 30 15-week-old *Cs*-pre-exposed pigs were randomly distributed into two groups (MOCK and VACC). A total of 12 pigs were then sacrificed at 28 dpv to analyze the vaccine-induced immune response pre-challenge. The remaining 18 pigs were further divided into three groups with n = 6 (MOCK, CHALL, VACC + CHALL), challenged according to their group allocation and sacrificed 49 dpv to analyze the vaccine-induced immune response post challenge.

The animal trial was carried out as depicted in Figure 1A. Thirty 15-week-old *Cs*-pre-exposed outbred pigs (all female) from the University-owned research farm were brought to the University of Veterinary Medicine Vienna. Rectal swabs were collected, and a *Chlamydiaceae* qPCR, followed by a *Cs*-specific qPCR assay was performed [36], confirming that all pigs showed rectal, but not vaginal, shedding of *Cs*. Pigs were then randomly distributed into the groups outlined in Figure 1B. After an acclimatization period of 7 days, pigs were administered 12.5 mg/kg body weight of doxycycline (Pulmodox 5%, pig premix for medicated feeding stuff, Virbac, France) daily orally for 8 days and, additionally, during the last 3 days, 10 mg/kg body weight of tylosin (Axentyl^®^ 200 mg/mL, Virbac, France) intramuscularly once a day to reduce *Cs* burden. After this, a resting period of 14 days was included to provide time for a potential anti-*Cs* immune response to decline. Analysis of rectal swabs using a *Cs*-specific qPCR confirmed that the pigs were negative for *Cs* after antibiotic treatment. At 0 days post (first) vaccination (dpv), pigs received the first intramuscular (IM) vaccination followed by a second IM vaccination 7 days later. The vaccine did not elicit any local adverse reactions at the injection site. At 14 dpv and 21 dpv, pigs received vaccinations intranasally (IN). The vaccinated group received CPAF adjuvanted with STING agonist (2′3′-c-di-AM(PS)2 (Rp,RP)) and the MOCK group received PBS (see Section 2.3). As outlined in Figure 1A, blood and vaginal swabs were collected every 7 days: 0, 7, 14, 21, 28 dpv. At 28 dpv, the pre-challenge necropsy took place with n = 6 MOCK and n = 6 VACC pigs to collect oviduct flushes. Pigs were anesthetized via intramuscular injection of Ketaminhydrochlorid (Ketamidor^®^, 20 mg/kg body weight, Vétoquinol, Lure Cedex, France) and Azaperon (Stresnil^®^, 40 mg/mL, 1–2 mg/kg body weight, Elanco, Greenfield, IN, USA), followed by euthanasia through intracardiac injection of T61^®^ (tetracaine hydrochloride, mebezonium iodide, and embutramide; 1.0 mL/10 kg body weight, MSD Animal Health, Kenilworth, NJ, USA). The remaining 18 pigs were divided into the following groups: MOCK (no vaccine, no challenge), CHALL (no vaccine, but challenged), and VACC + CHALL (vaccinated and challenged). To facilitate the trans-cervical challenge, estrous cycles were synchronized with the steroidal progestin altrenogest (Altresyn 4mg/mL Ceva Tiergesundheit, France) from 20 to 37 dpv. On day 38 dpv, 1000 international units of serum-Gonadotropin (Folligon^®^ 1000 I.U., MSD Tiergesundheit, Vienna, Austria) were injected intramuscularly, followed by a Choriongonadotropin (1500 I.U.; Chorulon^®^, MSD Tiergesundheit, Austria) injection 72 h later (41 dpv). A period of 24 h later (42 dpv), the cervix was open, and pigs were inoculated trans-cervically either with 20 mL sucrose phosphate glutamic acid (SPG) buffer (MOCK) or with 1 × 10^8^ inclusion-forming units (IFU) *Ct* serovar D in 20 mL SPG by inserting an intrauterine insemination catheter connected to an 80 mL semen tube. To monitor infection, pigs were swabbed cervically daily. On the day of the challenge (42 dpv) and one week after the challenge (49 dpv), blood samples and additional swabs were taken. Eight days after the challenge, pigs were sacrificed as described above to collect oviduct flushes and uterine swabs.

Blood samples were collected to isolate serum and peripheral blood mononuclear cells (PBMCs) for the analysis of systemic humoral and cell-mediated anti-CPAF immune responses, respectively. Swabs and oviduct flushes were used to analyze the local anti-CPAF antibody response. This animal trial was approved by the Ethics and Animal Welfare Committee of the University of Veterinary Medicine, Vienna, in accordance with the University’s guidelines for Good Scientific Practice and authorized by the Austrian Federal Ministry of Education, Science and Research (BMBWF 2023-0.588.126) in accordance with current legislation.

### 2.2. Vaccine Antigen Production and Formulation

The recombinant inactivated CPAF vaccine antigen was produced in *E. coli* as previously described [13]. Briefly, CPAF sequence is based on WP_015506580 using a codon-optimized open reading frame omitting the first 26 residues to facilitate expression, and proteolytic activity was compromised by including a S499 → A499 substitution. This sequence was then cloned into pRSETA. Following purification of the expressed protein, residual LPS was eliminated using cloud-point detergent extraction, and the CPAF protein was lyophilized for storage. The STING agonist (2′3′-c-di-AM(PS)2 (Rp,RP)) adjuvant was prepared according to manufacturer’s instructions (InvivoGen, San Diego, CA, USA). Within 1 h of vaccination, the following vaccine compositions were prepared in phosphate-buffered saline (PBS):

Per pig for the IM vaccine: 85 μg CPAF + 28.3 μg STING agonist

Per pig for the IN vaccine: 30 μg CPAF + 10 μg STING agonist

Afterwards, the vaccine was kept on ice until vaccine administration. For each vaccination site, the final vaccination volume was 1 mL per pig for IM vaccination and 2 mL per pig for IN vaccination (1 mL/nostril). Pigs in the MOCK group received the same volumes of PBS.

### 2.3. Cell Isolation, Swabs and Sera

PBMCs were isolated from heparinized blood using density gradient centrifugation with lymphocyte separation medium (Pancoll human, PAN Biotech, Aidenbach, Germany) and SepMate tubes (StemCell, Vancouver, BC, Canada), following the manufacturers’ instructions. Red blood cells were lysed using RBC lysis solution (Thermo Fisher Scientific, Waltham, MA, USA). After isolation, cells were counted using a Sysmex XP 300 hematology analyzer (Sysmex Europe GmbH, Norderstedt, Germany), and fresh PBMCs were used for in vitro restimulation to assess the anti-CPAF cytokine response via ELISpot (see Section 2.5). Remaining PBMCs were cryopreserved in freezing medium (50% RPMI 1640, 40% FBS, 10% DMSO) and stored at −80 °C for one day and then transferred to −150 °C. Remaining PBMCs were cryopreserved in freezing media (50% RPMI 1640, 40% FBS, 10% DMSO) and stored in −150 °C for future flow cytometric analysis. Serum samples were allowed to clot for over 30 min, centrifuged at 1500× *g* for 10 min, then aliquoted and stored at −20 °C for subsequent anti-CPAF antibody analysis.

Rectal swabs were collected by inserting swabs into the pig’s rectum. For vaginal swab collection, the vulva was cleaned, and the swab was gently rotated against the vaginal epithelium. For cervical swab collection following challenge, the vulva was cleaned, a speculum was inserted, and the swab was rotated against the cervical epithelium. For each location, two swabs were collected: one was placed in 1mL of PBS for antibody analysis, and one was placed in 1mL of sucrose phosphate (SP) buffer to monitor chlamydial burden. Tubes containing swabs and PBS were mixed by vortexing. Before the swab was removed from the tube, it was rotated against the tube wall. The samples were then frozen at −20 °C for future antibody analysis. The swabs in SP buffer remained in the buffer and were stored at −80 °C.

### 2.4. IFN-γ and IL-17A ELISpots

IFN-γ and IL-17 ELISpot assays were conducted according to the manufacturer’s instructions (MabTech, Nacka Strand, Sweden). Briefly, plates were pre-activated with ethanol and coated overnight at 4 °C with either anti-IFN-γ (pIFNγ-I, MabTech) or anti-IL-17A (MT49A7, MabTech) to capture antibodies. Fresh PBMCs were seeded at 0.25 × 10^6^ per well for IFN-γ ELISpots and 0.5 × 10^6^ cells per well for IL-17A ELISpots. Cells were stimulated for 48 h with 2 µg/mL CPAF. Medium alone and Concanavalin A (ConA, 3 µg/mL) served as negative and positive controls, respectively. Following incubation, cells were removed, and plates were incubated with the corresponding biotinylated detection antibodies: anti-IFN-γ (P2C11, MabTech) or anti-IL-17A (MTP853, MabTech). Signal development was performed using streptavidin–alkaline phosphatase and BCIP/NBT substrate (100 µL/well, Sigma-Aldrich, St. Louis, MO, USA). Plates were then dried, and spot-forming units were counted using an AID ELISpot reader (AID, Straßberg, Germany). Data represent the mean of three technical replicates.

### 2.5. In Vitro Stimulation and Flow Cytometry Staining

The CPAF-specific T cell response was evaluated with cryopreserved PBMCs. Before restimulation, thawed PBMCs were rested for 16 h at a cell concentration of 1 × 10^6^/mL. Then, PBMC were restimulated with 2 µg/mL CPAF to evaluate cytokine production or proliferation. For intracellular staining of IFN-γ and TNFα, round-bottomed 96-well microtiter plates (Greiner Bio One, Frickenhausen, Germany) were seeded with 5 × 10^5^ rested PBMCs in quadruplicates in cell culture medium: RPMI 1640 (PAN Biotech) supplemented with 10% fetal bovine serum (FBS, Merck KGaA, Darmstadt, Germany) and Penicillin-Streptomycin (PanBiotech). For cytokine production, PBMCs were either left in cell culture medium (negative control), or stimulated overnight with 2 µg/mL CPAF. Cells stimulated with 3 µg/mL of ConA served as positive controls. After 14 h of culture, Brefeldin A (BD GolgiPlug^TM^, BD Biosciences, San Jose, CA, USA) was added to the microcultures at a final concentration of 1 μg/mL for 4 h to block Golgi transport. Cells from four wells were then pooled and stained for flow cytometry as described below. In order to analyze T cell proliferation, rested PBMCs were stained with CellTrace^TM^ Violet (Thermo Fisher Scientific) according to manufacturer’s instructions and seeded in 96-well plates at a density of 2.5 × 10^5^ cells/well in cell culture medium. The CellTrace^TM^ Violet stained cells were either cultured in the presence of 2 µg/mL of CPAF or ConA (3 µg/mL; positive control) or left in media (negative control) for 4 days. After cultivation, quadruplicates were pooled and stained for flow cytometry as described below.

Stimulated PBMCs for the evaluation of CPAF-specific cytokine production or proliferation were surface-stained with primary monoclonal antibodies, including the directly conjugated antibodies as outlined in Table 1. The next staining step included secondary antibodies and the Fixable Viability Dye eFlour780 (Thermo Fisher) to discriminate dead cells according to manufacturer’s instructions. An additional intracellular staining was performed on the samples intended for the evaluation of CPAF-specific cytokine production, using the BD Cytofix/Cytoperm^TM^ Fixation/Permeabilization Kit (BD Biosciences, San Jose, CA, USA) according to the manufacturer’s instructions. All staining steps were carried out for 20min or 30min (intracellular staining) at 4 °C. Antibodies were titrated before their use, and compensation was calculated after measurement of single-color stained cells. Technical information about the antibodies used are listed in Table 1. At least 1 × 10^6^ lymphocytes per sample were collected on a Beckman Coulter CYTOFLEX LX^TM^ (laser configuration: U3-V5-B3-Y5-R3-I2). Data were analyzed with FlowJo^TM^ Software (Version 10.8.1; BD Biosciences) with gates based on the fluorescence minus one (FMO) controls. Cells were subjected to dead cell and doublet exclusion, as shown in Supplementary Figures S1 and S2. In addition to the percentage analysis of cell subsets, we calculated the “Differentiation value” as described in Amaral et al. (2020) [18]. Briefly, we assigned numerical values to the three differentiation statuses: naïve T cells = 0, T_CM_ = 1, and T_EM_ =2. Next, we multiplied the frequency of each differentiation status by its assigned numerical value and summed the products.

### 2.6. Anti-CPAF IgG and IgA ELISAs

Polystyrene 96-well plates (NUNC MaxiSorp, Thermo Fisher Scientific) were coated with 5 µg/mL of CPAF in a carbonate coating buffer at 100 μL/well. Coated plates were incubated overnight at 4 °C. The coated plates were then washed three times with wash buffer (PBS, 0.02% Tween 20), followed by a blocking step for at least 1 h at room temperature using 1% bovine serum albumin (BSA) in PBS. After four washes, diluted sera, swab eluate or oviduct flushes were added in duplicates and incubated overnight at 4 °C. For the evaluation of the systemic anti-CPAF antibody response, sera were diluted 1:5000 in assay buffer (PBS, 0.01% Tween-20, 0.1% BSA) prior to the addition to the plate. Samples for the local anti-CPAF antibody response (swab eluates and oviduct flushes) were used undiluted. Following the overnight incubation of samples, the plates were washed four times, and horseradish peroxidase-conjugated anti-pig IgA (A100.104P) or IgG (A100-117P) detection antibodies were added to the wells for 2 h at room temperature (Bethyl Laboratories Inc., Montgomery, TX). After four washes, substrate (3,3′,5,5′-Tetramethylbenzidine (TMB)) was added and incubated for 30 min at room temperature, followed by the addition of sulphuric acid to stop the color reaction. The optical density (OD) of the reaction product was quantified by measurements at 450/620 nm using a Tecan Sunrise ELISA reader (Tecan, Männedorf, Switzerland). For the IgG avidity assessment, the protocol was modified: a 6 M urea treatment was added after the sample incubation. After incubation with serum samples, the plates were washed four times and then incubated in assay buffer (control treatment) or 6 M urea for 10 min at room temperature, followed by four washes and the addition of TMB substrate. The avidity index was determined by dividing the OD value of the 6 M urea-treated sample by the OD value of the control sample.

### 2.7. Detection of Chlamydiaceae via qPCR

DNA was extracted using the QIAmp DNA Mini Kit (Qiagen, Hilden, Germany) following the manufacturer’s instructions, with an elution volume of 100 μL. The extract was analyzed on a qTower3 Real-Time PCR Thermocycler (Analytik Jena, Jena, Germany) using a *Chlamydiaceae* family-specific PCR targeting the 23S rRNA gene, with an internal amplification control as previously described by Blumer et al. [37]. A sixfold dilution series of *C. abortus* DNA was used to generate the standard curve. A positive reference material served as the positive control, while DNA-free water was used as the negative control. The threshold line was set at 0.1 for all samples in each run. Data are presented as 42 – Ct value.

### 2.8. Statistical Analysis

Data were analyzed using GraphPad Prism 10.4.2 software. Prior to applying parametric statistical tests, normality was assessed using the Shapiro–Wilk test, with a *p*-value ≥ 0.05 indicating a normal distribution. Depending on the data, two-way ANOVA, one-way ANOVA, or the Mann–Whitney U test was used, as specified in the figure legends. For significant results from two-way or one-way ANOVA, Tukey’s post hoc test was performed for pairwise comparisons. The levels of significance were defined as *p* ≤ 0.05 (*), *p* ≤ 0.01 (**), and *p* ≤ 0.001 (***).

## 3. Results

**Figure 2:**
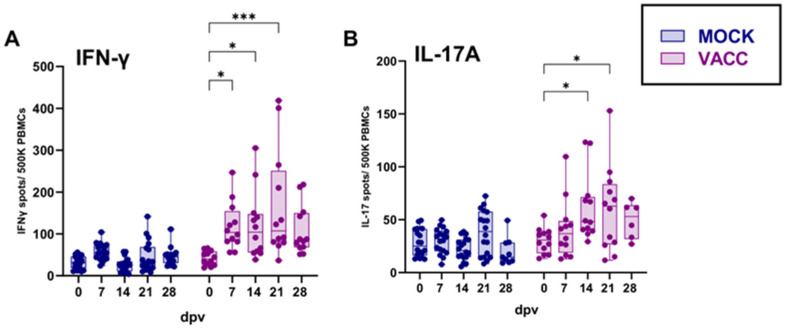
IFN-γ and IL-17A are produced by PBMCs from vaccinated pigs in response to in vitro CPAF restimulation. (**A**) IFN-γ production by freshly isolated PBMCs was measured by ELISpot after overnight in vitro CPAF restimulation. (**B**) IL-17A production by freshly isolated PBMCs, which was also measured by ELISpot upon overnight in vitro CPAF restimulation. Each symbol represents an individual animal (n = 18 Mock and n = 12 Vaccinated). Due to technical difficulties, the sample size at day 28 had (IL-17A) to be reduced to n = 9 Mock and n = 6 Vaccinated. The statistical analysis of within-group comparisons is shown. Statistical analysis was performed using two-way ANOVA followed by Tukey’s multiple comparisons test. * *p* < 0.05, *** *p* < 0.001. dpv = days after first vaccination.

**Figure 3:**
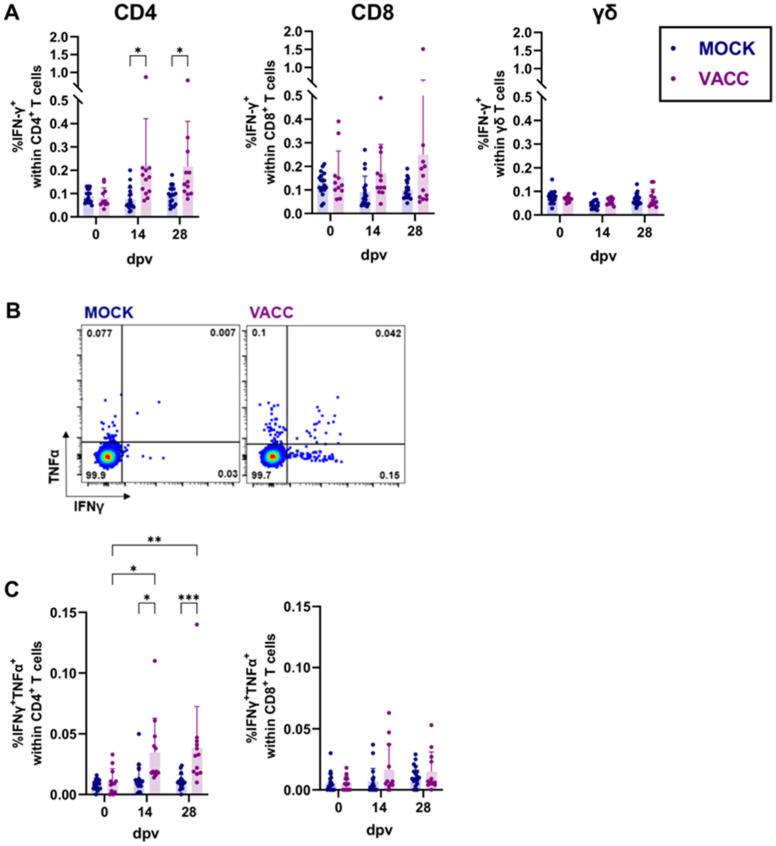
IFN-γ and TNF-α production by T cell subsets in response to in vitro CPAF restimulation. Cryopreserved PBMCs were thawed, rested, and restimulated in vitro with CPAF. During data analysis, dead cells and doublets were excluded, as shown in Supplementary Figure S1. After identification of T cell subsets, their IFN-γ and TNF-α production was analyzed. (**A**) The scatter diagrams show the percentage of IFN-γ positive cells with CD4, CD8 of γδ T cells at different time points. (**B**) Representative plots for CPAF-specific cytokine production are shown. The frequency of IFNγ^+^TNFα^+^ cells within CD4 and CD8 T cells is shown in (**C**). Each symbol represents data from one individual pig (n = 18 for Mock and n = 12 for Vaccinated). The statistical analysis was performed via GraphPad using 2-way ANOVA and Tukey multiple comparisons test. * *p* < 0.05, ** *p* < 0.01, *** *p* < 0.001. dpv = days after first vaccination.

**Figure 4:**
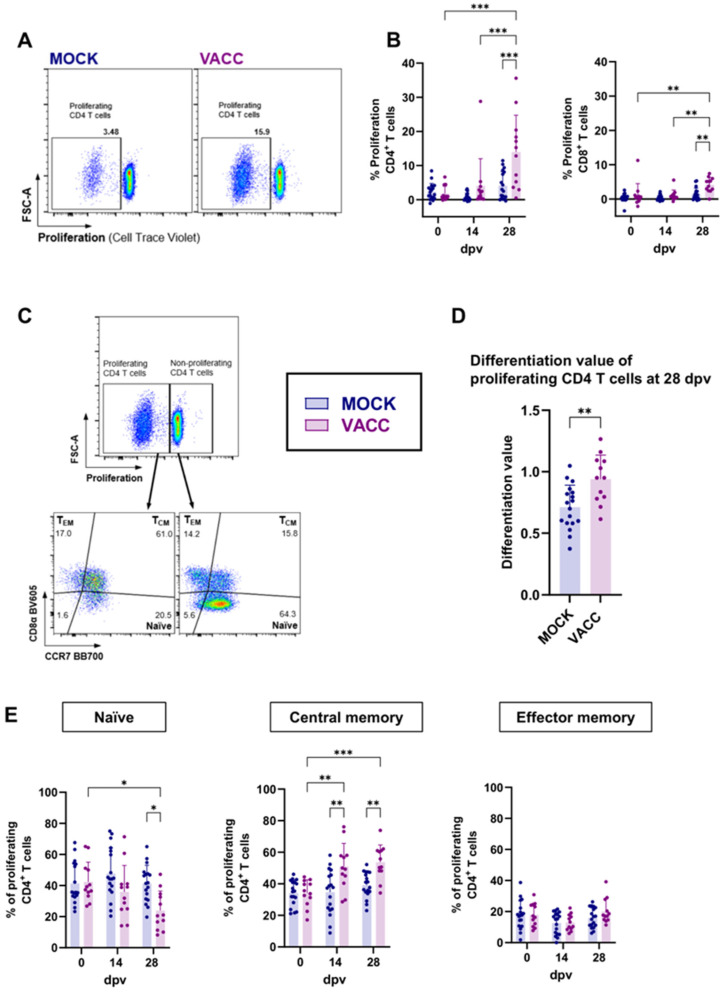
Proliferation of CD4 and CD8 T cells and differentiation of proliferating CD4 T cells. Previously cryopreserved PBMCs were thawed, rested, and stained with CellTrace^TM^ Violet before being cultured with CPAF for 4 days. Cells were then harvested and stained as indicated in Table 1. During data analysis, dead cells and doublets were excluded, as shown in Supplementary Figure S2. (**A**) Representative plots for CPAF-specific proliferation of CD4 T cells at 28 days after (first) vaccination (dpv) are shown. (**B**) shows the proliferative response of CD4 and CD8 T cells according to their vaccination groups—MOCK (blue) and VACC (purple) over time. Shown data were background corrected by subtracting the % proliferation in media (negative control, background) from the % proliferation under CPAF stimulation. Panel (**C**) shows the gating hierarchy to assess the differentiation of proliferating CD4 T cells. After gating on proliferating CD4 T cells, their differentiation was analyzed via their CCR7/CD8a expression profile to distinguish naïve (CCR7^+^CD8α^−^), central memory (TCM, CCR7^+^CD8α^+^) and effector memory (TEM, CCR7^−^CD8α^+^) CD4 T cells. Differentiation values were calculated for proliferating CD4 T cells (**D**). Panel (**E**) shows the frequency of the naïve, TCM and TEM subsets within proliferating CD4 T cells according to their vaccination groups and over time. Each symbol represents data from one individual pig (n = 18 for Mock and n = 12 for Vaccinated). The statistical analysis was performed via GraphPad using 2-way ANOVA and Tukey multiple comparisons test (**B**,**E**) or Mann–Whitney U test (D). * *p* < 0.05, ** *p* < 0.01, *** *p* < 0.001.

**Figure 5:**
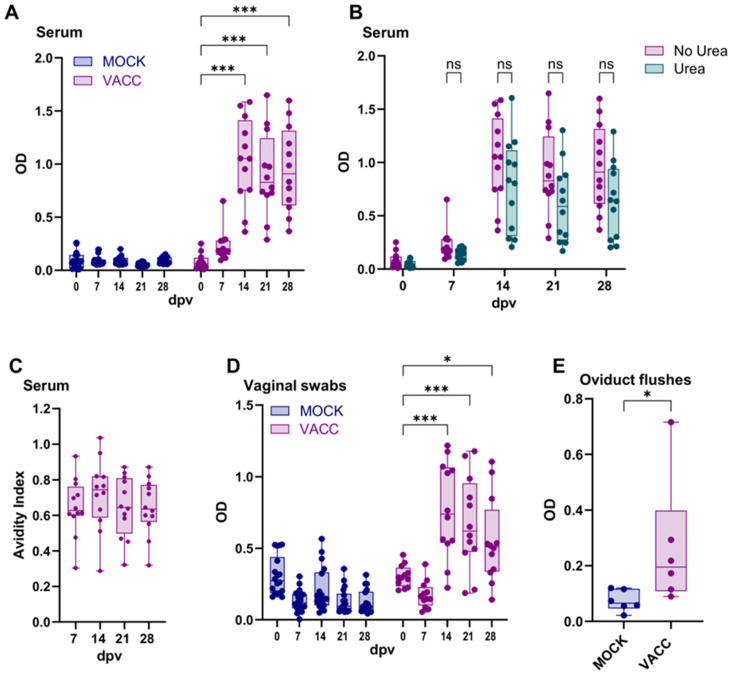
Systemic and local anti-CPAF IgG levels and avidity. (**A**) Immunoglobulin G (IgG) levels were quantified by anti-CPAF IgG ELISA in sera from MOCK and VACC animals. Data show optical density (OD) values. (**B**) shows OD values before and after 6M urea treatment, which was used to assess the IgG avidity. (**C**) shows the avidity index values which are calculated by dividing the OD value of the 6M urea-treated sample by the OD value of the control-treated samples. (**D**,**E**) IgG levels were quantified by anti-CPAF IgG ELISA in vaginal swabs (**D**) from MOCK and VACC animals throughout the trial and in oviduct flushes (**E**) from pigs euthanized at day 28. Each symbol represents data from one individual pig (n = 18 for Mock and n = 12 for Vaccinated; n = 6 Mock and n = 6 Vaccinated in (**E**)). The statistical analysis was performed via GraphPad using 2-way ANOVA and Tukey multiple comparisons test (**A**,**B**,**D**) or one-way ANOVA and Tukey multiple comparisons test (**C**) or Mann–Whitney U test (**E**). * *p* < 0.05; *** *p* < 0.001. dpv = days after first vaccination.

**Figure 6:**
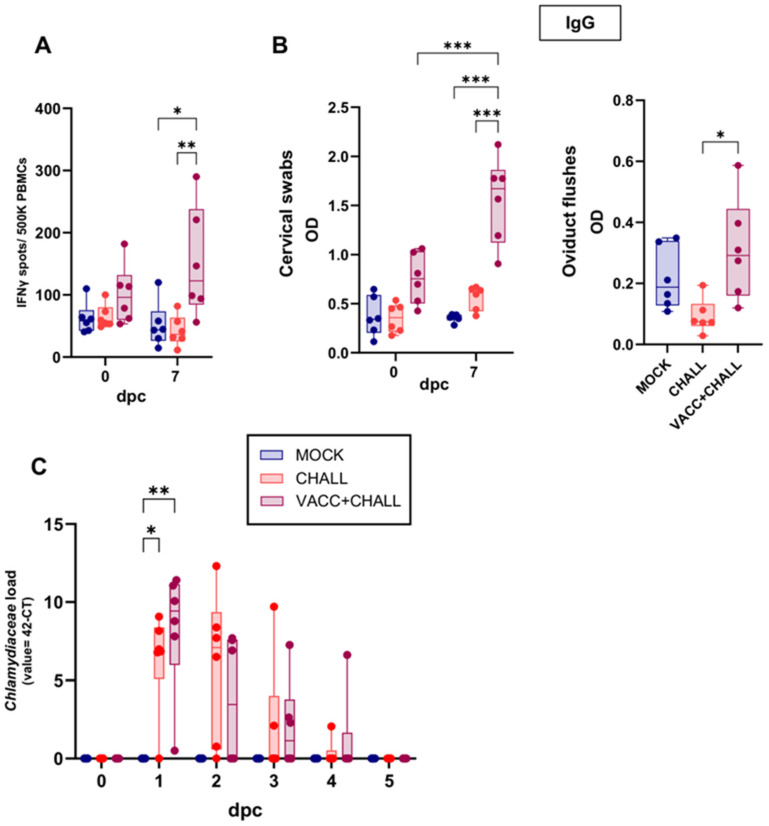
Vaccinated pigs show a local IgG response post challenge but their *Chlamydiaceae* load is not reduced. Pigs were challenged with *C.trachomatis* 6 weeks after the first vaccination (42 dpv). Pre-challenge and 7 days post challenge (dpc), the systemic IFN-γ response (**A**) and the local IgG response (**B**) was measured. (**A**) IFN-γ production by PBMCs was measured by ELISpot after overnight in vitro CPAF restimulation of freshly isolated PBMCs from MOCK (blue), *C. trachomatis* challenged (CHALL, orange), vaccinated + *C. trachomatis* challenged (VACC + CHALL, red) pigs. (**B**) IgG levels were quantified by anti-CPAF IgG ELISA in cervical swabs and oviduct flushes. (**C**) *Chlamydiaceae* load was analyzed via qPCR in cervical swabs prior to challenge (dpc) and daily after challenge (1–5 dpc). Each symbol represents data from one individual pig (n = 6 per group). The statistical analysis was performed via GraphPad using 2-way ANOVA and Tukey multiple comparisons test or one-way ANOVA with Tukey multiple comparisons test (B, oviduct flushes). Only statistically significant comparisons are shown. * *p* < 0.05; ** *p* < 0.01; *** *p* < 0.001.

*Cs*-pre-exposed pigs were used to study vaccine immunogenicity and efficacy after 2× IM and 2× IN vaccinations with the CPAF/c-di-AMP vaccine candidate as outlined in Figure 1. Vaccine immunogenicity was assessed by IFN-γ and IL-17A ELISpots (Figure 2), IFN-γ and TNF-α flow cytometric intracellular cytokine staining (Figure 3), proliferation analysis (Figure 4), and anti-CPAF ELISAs (Figure 5). Vaccine efficacy was evaluated after trans-cervical *Ct* challenge and subsequent assessment of *Chlamydiaceae* burden and local IgG response (Figure 6).

### 3.1. Vaccination with CPAF/c-di-AMP Elicits a Systemic IFN-γ and IL-17A Response

The vaccine-induced CPAF-specific systemic IFN-γ and IL-17A responses were assessed via ELISpot assays. In contrast to MOCK-vaccinated pigs, the vaccinated group showed a slight but measurable increase in IFN-γ and IL-17A production in response to CPAF restimulation (Figure 2). The IFN-γ production was significantly increased as early as 7 dpv (mean 113 spots/500K PBMCs). Thereafter, no further increase was observed (Figure 2A). A similar pattern was noted for the IL-17A production: while PBMCs from MOCK-vaccinated pigs produced IL-17A at background levels, PBMCs from vaccinated pigs released IL-17A from 14 dpv onwards (Figure 2B). It must be noted that due to technical difficulties, the sample size at 28 dpv had to be reduced for the IL-17A ELISpot assay to n = 9 for MOCK and n = 6 vaccinated pigs. In summary, the vaccine induced low-level CPAF-specific systemic IFN-γ and IL-17A production.

### 3.2. CPAF/c-di-AMP Induces Cytokine Production, Proliferation, and Differentiation in CD4 T Cells

Since we observed a vaccine-induced systemic cytokine response via ELISpot assays (Figure 2), we further investigated the CPAF-specific T cell response using flow cytometry. This analysis included CPAF-specific cytokine production (Figure 3), proliferation, and the formation of central and effector memory T cells (Figure 4). Cryopreserved PBMCs were thawed, rested, restimulated in vitro with CPAF, and stained for flow cytometry analysis using antibodies and fluorochromes listed in Table 1. The gating strategy is shown in Supplementary Figure S1 (cytokine production) and Supplementary Figure S2 (proliferation). As expected, the frequency of IFN-γ^+^ cells did not increase over time in MOCK-vaccinated pigs (blue, Figure 3A). In line with the moderate cytokine response noted in the ELISpot assays, the frequency of IFN-γ^+^ CD4 T cells was only slightly but significantly higher in vaccinated pigs at 14 and 28 dpv (purple, Figure 3A). This increase in IFNγ^+^ cells was not evident within CD8 T cells or γδ T cells (Figure 3A). In addition to the investigation of IFN-γ production by T cell subsets, we included an analysis of TNF-α. Although the CPAF/c-di-AMP immunization had no effect on the frequency of TNF-α^+^ single-positive cells (Supplementary Figure S3), it led to an increase in multifunctional IFN-γ^+^TNF-α^+^ CD4 T cells (Figure 3B,C) at 14 and 28 dpv. However, the overall frequency of these multifunctional T cells remained low.

Since antigen-specific proliferation is a crucial function of memory T cells, we utilized it as an additional measure of vaccine immunogenicity and T cell function. PBMCs were restimulated in vitro with CPAF for 4 days before antigen-specific proliferation as well as the differentiation of responding T cells was analyzed (Figure 4). Gamma-delta T cells showed no CPAF-specific proliferation in vaccinated pigs compared to MOCK animals for all time points tested (Supplementary Figure S4A). The proliferative response of CD4 and CD8 T cells to CPAF restimulation is shown in Figure 4A,B. While raw proliferation data are shown in Supplementary Figure S4B, data shown in Figure 4B were background corrected by subtracting the % background proliferation in media (negative control) from the % proliferation under CPAF stimulation. In control pigs, CPAF-specific proliferation of CD4 and CD8 T cells remained low at all time points. In vaccinated pigs, proliferation also remained low before and after 14 dpv. At 28 dpv, however, the proliferative response in vaccinated pigs increased significantly for both CD4 and CD8 T cells: at this time point, up to 35% of CD4 T cells and up to 6.5% of CD8 T cells from vaccinated pigs exhibited a proliferative response (Figure 4B).

The CD4 T cells responding to CPAF restimulation with proliferation were additionally analyzed for differentiation from naïve over central memory (T_CM_) or effector memory (T_EM_) phenotypes (gating strategy shown in Figure 4C). At the peak of CD4 T cell proliferation (28 dpv), we used a strategy to quantify T cell differentiation described by Amaral et al. [18] as “differentiation value” (Figure 4D). As described in Materials and Methods, this differentiation value assesses the differentiation status of the responding cells by considering their distribution over naïve, T_CM_ and T_EM_ phenotypes. This analysis showed that, compared to MOCK pigs, proliferating CD4 T cells from c-di-AMP-CPAF-vaccinated pigs were significantly more differentiated at 28 dpv (Figure 4D). More specifically, while in MOCK pigs about half (mean 41.3%) of proliferating T cells were naïve, only about 24% of proliferating T cells had a naïve phenotype in CPAF-vaccinated pigs at 28 dpv (Figure 4E). In vaccinated pigs, the predominant differentiation status of proliferating CD4 T cells was T_CM_, comprising 51–54% at 14 and 28 dpv (Figure 4E). Notably, CPAF-specific CD4 T_EM_ cells exhibited no increased proliferative response across all groups and time points measured. Overall, these data show that the CPAF/c-di-AMP vaccine candidate induces a robust proliferative CD4 T cell response with a higher frequency of central memory differentiated CD4 T cells.

### 3.3. The CPAF/c-di-AMP Vaccine Induced Systemic and Local IgG Responses

Although the role of antibodies during a Ct infection and resolution thereof is still unclear, we evaluated the systemic and local antibody responses via ELISAs as another measure of adaptive immunity. The systemic antibody response was assessed by measuring anti-CPAF IgG levels in serum samples using ELISA (Figure 5A). While the serum IgG levels in MOCK pigs stayed low throughout the trial, the anti-CPAF serum IgG levels in vaccinated pigs increased significantly between 7 and 14 dpv. Thereafter, IgG levels remained constant (Figure 5A). In addition to antibody levels, anti-CPAF IgG antibodies in vaccinated pigs were further assessed for avidity. As shown in Figure 5B, the avidity index was already at a generally high level at 7 dpv (mostly 0.6–0.8). This value did not increase over time (Figure 5C). This indicates that in most anti-CPAF-vaccinated animals, over 60% of antibodies exhibited strong avidity to the Ct CPAF vaccine antigen. However, no further antibody maturation was observed within the four weeks following the initial vaccination.

In addition to analyzing the systemic IgG response, we evaluated the mucosal humoral response to CPAF via IgG and IgA ELISAs in vaginal and rectal swabs, as well as in oviduct flushes (Figure 5D,E; Supplementary Figure S5A–C). Similarly to the anti-CPAF IgG levels in serum, an increase in anti-CPAF IgG was observed in vaginal swabs at 14 dpv in vaccinated pigs (Figure 5D). This increase was also statistically significant compared to the MOCK group. After 14 dpv, a slight but non-significant decrease in IgG levels was noted in the vaccinated group. Rectal anti-CPAF IgG was detected at lower levels and only increased at 14 dpv in 3 of 12 vaccinated pigs (Supplementary Figure S5A). IgA levels in both vaginal and rectal swabs did not reveal vaccine-induced differences, and higher variability between individual animals was observed (Supplementary Figure S5B). At necropsy, anti-CPAF IgG and IgA levels were also quantified in oviduct flushes collected from n = 6 per group at 28 dpv to investigate the humoral immune response in the upper genital tract. While the IgA response of vaccinated pigs was low (OD < 0.1, Supplementary Figure S5C), these pigs demonstrated higher and significantly elevated anti-CPAF IgG levels in oviduct flushes (Figure 5E). Taken together, vaccination with the CPAF/c-di-AMP vaccine candidate induced a robust serum anti-CPAF IgG response, along with mucosal anti-CPAF IgG responses in both the lower and upper genital tract.

### 3.4. CPAF/c-di-AMP-Vaccinated Pigs Are Not Protected from Ct Challenge but Show a Boosted Systemic CPAF-Specific IFN-γ and Local CPAF-Specific IgG Response

As outlined in Figure 1A, pigs were challenged trans-cervically 42 dpv with SPG buffer or *Ct*. The groups for this challenge were defined as follows: MOCK (mock-vaccinated and mock-challenged); CHALL (mock-vaccinated, *Ct*-challenged); and VACC + CHALL (CPAF/c-di-AMP-vaccinated, *Ct*-challenged). Vaginal swabs were taken daily post challenge to assess *Ct* shedding. On 0 and 7 dpc, blood and vaginal swabs were obtained to evaluate the systemic cell-mediated immune response and the mucosal humoral response before and after challenge. The systemic cell-mediated immune response was measured via IFN-γ ELISpot: while MOCK and CHALL pigs exhibited only background-level IFN-γ production with no increase post challenge, VACC + CHALL pigs showed an increase in IFN-γ production in 3 out of 6 VACC + CHALL pigs at 7 dpc. This increase was significant compared to both the MOCK and CHALL groups (Figure 6A).

While the rectal anti-CPAF IgG and genital anti-CPAF IgA responses were not altered significantly by Ct challenge (Supplementary Figure S6A–C), the genital anti-CPAF IgG response was noteworthy (Figure 6B): while MOCK and CHALL IgG levels remained constant before and after challenge, the VACC + CHALL group had higher anti-CPAF IgG levels in vaginal swabs and oviduct flushes. The increase in anti-CPAF vaginal swab IgG levels was not only significant compared to the MOCK and CHALL groups at 7 dpv, but also compared to the pre-challenge IgG levels of the VACC + CHALL group. This demonstrates that only CPAF/c-di-AMP-vaccinated animals reacted with local IgG production to CPAF after *Ct* challenge.

The effect of vaccination on the genital *Chlamydiaceae* load is shown in Figure 6C. Prior to challenge, pigs from all groups were negative for *Chlamydiaceae*. In addition, all MOCK-challenged animals (blue) stayed negative throughout the study. With one exception per group, pigs in the CHALL and VACC + CHALL groups were all positive for *Chlamydiaceae* as of 1 dpc. The chlamydial load peaked at 1 dpc with a slow decline thereafter. All pigs cleared *Chlamydiaceae* by 5 dpc. We did not observe any differences in the peak or clearance rate between the two challenged groups (CHALL and VACC + CHALL). Thus, despite the increase in cellular responses and mucosal IgG to CPAF in the vaccinated group post challenge, no vaccine-induced reduction in *Chlamydiaceae* load was evident.

## 4. Discussion

STING agonists, such as c-di-AMP, are considered ideal vaccine adjuvants for controlling intracellular pathogens, especially when strong Th1 and Th17 immune responses are crucial, as reviewed in [38]. Recently, the immunogenicity and efficacy of a CPAF/c-di-AMP vaccine candidate was tested against *Cm* infection: it demonstrated promising T cell immunogenicity and reduced chlamydial burden [35]. However, the potential of STING agonists as adjuvants has not yet been evaluated for *Ct* vaccines in the translational pig model. Thus, we aimed to determine the vaccine immunogenicity and efficacy of a CPAF/c-di-AMP vaccine candidate in our *Cs*-pre-exposed pig model.

We found that vaccination with CPAF/c-di-AMP induced a low-level systemic IFN-γ response by 7 dpv and IL-17A response by 14 dpv. Flow cytometry revealed that CPAF-specific cytokine production was primarily observed in CD4 T cells. The lack of a strong CD8 T cell response may be due to the use of whole protein for restimulation, which relies on cross-presentation by PBMCs and can result in an underestimated CD8 T cell response, although at low frequencies, vaccinated pigs exhibited an increase in multifunctional IFN-γ^+^TNF-α^+^ CD4 T cells. Especially, compared to the findings of Proctor et al. (2024) [13], where pigs vaccinated with TriAdj (PCEP, host defence peptides and poly(I:C)) adjuvanted CPAF exhibited a far stronger systemic cell-mediated immune response, the response induced by CPAF/c-di-AMP vaccination appears relatively weak. It should be noted that while the current study utilized 15-week-old pigs, Proctor et al. vaccinated 7-week-old pigs. Although the IM CPAF dosage was adjusted for body weight, the STING pathway agonist c-di-AMP appears to induce a weaker systemic cytokine response compared to the TriAdj adjuvant [13]. Notably, the older pigs utilized in the current study also had a longer *Cs* pre-exposure, which could have altered the anti-chlamydial immune response, resulting in reduced immune responses. Moreover, a limitation of the current study is the absence of a group receiving c-di-AMP alone, which restricts our ability to fully access the adjuvant’s independent contribution to the observed immune responses. While the TriAdj adjuvant has been successfully used via the intramuscular route in various species, the STING agonist c-di-AMP has primarily been studied as a mucosal adjuvant [39,40,41,42,43]. Ebensen and colleagues (2019) conducted one of the few studies investigating c-di-AMP as a parenteral (IM) adjuvant in mice using the model antigen β-Gal [44]. While cellular responses, including proliferation and cytokine production, were detectable in the β-Gal + c-di-AMP group, a significant production of Th1, Th17 and Th2 cytokines was only observed when c-di-AMP was combined with alum. This finding highlights that c-di-AMP alone was insufficient to induce a robust systemic cellular immune response through the parenteral route. Similarly, studies on c-di-GMP and cGAMP, both cyclic dinucleotides and STING pathway agonists, indicate that these adjuvants exhibit poor efficacy when administered intramuscularly [45,46]. One possible explanation is the rapid diffusion of cGAMP, and potentially other cyclic dinucleotides, from the inoculation site. A comparison of cGAMP concentration in skin and muscle after intradermal (ID) or IM injection, respectively, revealed that cGAMP was sustained at higher concentration and for a longer time in the skin. In muscle tissue, cGAMP levels dropped sharply within the first three hours post injection, leaving only a brief window for the recruitment of immune cells and activation of antigen-presenting cells (APCs) [46]. Hence, it is possible that the IM-administered cyclic dinucleotide c-di-AMP disseminated too quickly from the injection site before a robust activation of APCs was possible, consequently resulting in the weak systemic cytokine response observed in this study. Moreover, cyclic dinucleotides display poor membrane permeability due to the presence of negatively charged phosphate groups and are rapidly degraded by nucleotide hydrolases within the cell resulting in limited stability [47,48,49]. Future work will aim to limit the diffusion of a STING agonist by directly conjugating it to the antigen via click chemistry to extend its local exposure to antigen-presenting cells; this might enhance the CPAF/c-di-AMP immunogenicity. In addition, ID administration might be a promising option to deliver the vaccine candidate directly to the plethora of antigen-presenting cells in the skin. In addition, formulations that enhance the intracellular delivery of cyclic dinucleotides may improve the overall efficacy of these adjuvants.

While the quick dissemination could explain limited IM-based vaccine immunogenicity, it does not necessarily explain the lack of an improved cytokine response after IN boosts (14 and 21 dpv). Following the IN administration of CPAF/c-di-AMP, the systemic cytokine response (IFN-γ, IL-17A, TNF-α) was not increased any further. This contrasts with studies in mice, where the IN administration of c-di-AMP adjuvanted vaccines as prime and boost resulted in strong systemic IFN-γ/IL-17A responses [35,50,51,52]. Although the vaccine was easily administered intranasally to pigs, it is possible that the pig’s nasal mucus layer or epithelium may have limited its penetration. It should also be noted that a transient or weak systemic cell-mediated immune response following IN delivery does not necessarily imply the absence of an immune response: the vaccine may have induced a local, mucosal immune response. Specifically, the generation of tissue-resident memory T cells (T_RM_ cells) has been linked to the mucosal administration route [53]. In the context of *Chlamydia* infections, T_RM_ cells appear to be essential for pathogen clearance. A study utilizing congenic (CD45.1/CD45.2) mice with a shared circulatory system (parabiosis) has shown that mice lacking T_RM_ cells exhibit only partial protection, even in the presence of circulating memory cells [54]. Moreover, intranasal/intrauterine UV-*Ct*–cSAP vaccination led to seeding of the uterine mucosa with T_RM_ cells within one-week post vaccination; yet, subcutaneous administration did not. This suggests that protective T_RM_ cells are inducible in mucosa-associated lymphoid tissue but not in skin-draining lymph nodes [54]. Although challenging, future studies on *Ct* vaccination should conduct a comprehensive analysis of mucosal immunity to better assess vaccine immunogenicity in the context of IN administrations.

While we observed an overall weak systemic IFN-γ production by CD4 T cells, we noted a significant proliferative response of CD4 T cells in vaccinated pigs at 28 dpv. T cell proliferation and IFN-γ production can occur independently. This is especially true when analyzing different T cell memory subpopulations such as T_EM_ and T_CM_. After TCR stimulation, T_CM_ secretes IL-2 and proliferates but does not immediately produce effector cytokines, whereas T_EM_ rapidly produces effector cytokines such as IFN-γ [55]. In our study, proliferating CD4 T cells from vaccinated pigs predominantly exhibited a T_CM_ phenotype. While we did not investigate IL-2 production or the differentiation status of IFN-γ-producing CD4 T cells, it is likely that IL-2 production occurred in the T_CM_ subset and IFN-γ production was facilitated by T_EM_ cells. Overall, the CPAF/c-di-AMP vaccine candidate seems to favor the induction of a CD4 T_CM_ memory response biased towards proliferation. Notably, IFN-γ-producing CPAF-specific T cells detected in women previously infected with *Ct* were primarily of the T_CM_ phenotype [27].

The role of anti-*Ct* antibodies in pathogen clearance and protection remains uncertain. Murine studies indicate a supportive role for antibodies in protection against chlamydia reinfection; adoptive transfer of immune serum also protects recipient mice against primary challenge [26,56,57,58]. However, this may only apply when the antibodies are directed towards the outer membrane proteins of chlamydia, like the major outer membrane protein (MOMP). Since CPAF is not associated with the infectious elementary bodies but secreted into the host cytosol, anti-CPAF antibodies may not directly affect the ability of chlamydia to infect host cells. Hence, anti-CPAF antibodies might not be able to provide protection against *Ct* infection. Indeed, Poston and colleagues (2024) observed that B-cell-deficient mice vaccinated with chimp adenovirus-vectored CPAF were still able to decrease chlamydial burden and shorten the course of *Cm* infection [59]. Similarly, B-cell-deficient mice vaccinated with CpG adjuvanted CPAF showed comparable *Cm* resolution kinetics to vaccinated WT mice [60]. Nevertheless, CPAF-specific antibodies were detected in 75% of *Ct*-infected individuals and can neutralize the proteolytic activity of CPAF in a cell-free degradation assay [61,62]. The significance of this prevalence and neutralization is still unclear, considering the cytosolic localization of CPAF until host cell lysis occurs. However, antibodies might be able to neutralize the extracellular activity of CPAF. CPAF has been reported to prevent activation of neutrophils, inhibit the alternative complement activation pathway, and degrade antimicrobial peptides [29,63,64]. Other effector mechanisms of anti-CPAF antibodies, like Fc-mediated antigen presentation and phagocytosis or complement activation, could also pay a role in enhancing cellular responses but have not been comprehensively explored yet. Despite the weak Th1 response, we show that the CPAF/c-di-AMP vaccine candidate induced a robust humoral response. Unexpectedly, antibody avidity was already high at 7 dpv and remained stable thereafter, suggesting that vaccination may have activated memory B-cells and long-lived plasma cells that had already undergone somatic hypermutation and antibody maturation. As a result, further antibody refinement was limited. This may be due to the pigs being pre-exposed to *Cs* and relatively mature (4 months old) at the time of study enrollment. Another reason for the lack of avidity increase could be that the vaccine induced poor T follicular helper cell support or that the soluble protein and adjuvant were cleared too fast. It is widely accepted that different adjuvants lead to different germinal center and antibody kinetics [65]; however, the exact mechanisms are not well understood. In addition to analyzing the systemic immune response, antibody levels in mucosal samples, such as vaginal/rectal swabs and oviduct flushes were measured. While no IgA responses could be detected, the level of anti-CPAF IgG increased significantly post vaccination in the lower and upper genital tract. Thus, the CPAF/c-di-AMP vaccine candidate resulted in an IgG-biased local humoral immune response. Overall, it remains to be seen if anti-CPAF antibodies, especially high-avidity ones, can protect against *Ct*.

To evaluate vaccine efficacy, pigs were challenged at 42 dpv trans-cervically with *Ct*. In accordance with Käser et al. (2017) and Lorenzen et al. (2017), *Ct* can be detected in vaginal swabs for a few days post infection [17,66]. Despite a short infection period following chlamydia challenge in pigs, the impact of vaccination remained measurable. As demonstrated by Amaral et al. (2020), compared to unvaccinated pigs, pigs vaccinated with UV-inactivated *Cs* exhibited, at 2 and 3 days post challenge, a significantly lower genital *Cs* burden [18]. However, in the current study, the peak chlamydial burden was already observed one day after challenge followed by a constant decline. This kinetic pattern may indicate either a transient infection or only the detection of administered *Chlamydia*. With this limitation in mind, we did not observe a difference between the mock-vaccinated and challenged (CHALL) and the CPAF/c-di-AMP-vaccinated and challenged (VACC + CHALL) pigs in terms of chlamydia peak or clearance rate. Although there was no significant difference in chlamydial burden between vaccinated and unvaccinated groups, the vaccinated animals exhibited significantly stronger CD4 T cell and local IgG responses after challenge. It should be noted that the experimental challenge dose of 10^8^ IFU is a high dose and likely exceeds the estimated infectious dose of *Ct* encountered during natural sexual transmission in humans. Dirks and colleagues (2015) used a qPCR-based assay to quantify the *Ct* load in infected women and found a median *Ct* load of 1.7 × 10^5^/mL in vaginal swabs [67]. Semen from *Ct*-infected males has been found to contain up to 1.6 × 10^4^ *Ct* DNA copies per mL [68]. Although these studies did not measure the infectious dose directly, these estimates suggest that the infectious dose during natural exposure is likely much lower than the 10^8^ IFU used in our experimental settings. This could result in the failure to detect protection due to an excessively high experimental challenge.

In summary, while the *Ct* challenge part of the experiment did not conclusively answer the question of vaccine efficacy, it at least demonstrates effective local immune priming by the CPAF/c-di-AMP vaccine candidate.

## 5. Conclusions

In conclusion, the *Ct* CPAF/c-di-AMP vaccine candidate evaluated in this study elicited both cell-mediated and humoral immune responses in our *Cs*-pre-exposed outbred pig model. However, the systemic Th1 response was weaker compared to the previously tested TriAdj-adjuvanted CPAF vaccine candidate [13]. Despite an increase in mucosal antibody responses post challenge, the low level of vaccine-induced cellular immune response and limited role of anti-CPAF antibodies make us question the ability of this vaccine candidate to induce protection against *Ct* challenge. This underscores the need for further optimization of this vaccine candidate: potential improvements can be achieved by direct conjugation of the STING agonist to the CPAF protein through click chemistry and/or an alternate route of delivery (ID). Moreover, given the complexity of the chlamydial life cycle and its multiple developmental stages, an effective vaccine will likely need to be multivalent to elicit robust humoral and cellular immune responses. The combination of CPAF, a secreted chlamydial protein, with an outer membrane protein (e.g., MOMP) is a promising future direction.

This relevant pig model can further promote these optimization processes. Future studies in this model will assess the proposed optimized vaccine candidates and include a more detailed analysis of the local T cell responses.

## Figures and Tables

**Table 1:** Primary antibodies and secondary reagents used for flow cytometric analysis.

Antigen	Clone	Isotype	Fluorochrome	Labeling Strategy	Primary Antibody Source	Secondary Antibody Source
***Anti-CPAF T cell response: Cytokine production***						
CD3	PPT3	mIgG1	FITC	Directly conjugated	Southern Biotech (Birmingham, AL, USA)	-
CD4	74-12-4	mIgG2b	BV421	Secondary antibody	In house	Jackson ImmunoResearch (West Grove, PA, USA)
CD8α	76-2-11	mIgG2a	BUV395	Biotin-Streptavidin	Thermo Fisher	Biolegend
TCRγδ	PGBL22A	mIgG1	AF647 *	Directly conjugated	Kingfisher Biotech (Saint Paul, MN, USA)	Thermo Fisher
Live/Dead	-	-	eFlour780	-	Invitrogen (Waltham, MA, USA)	-
TNFα	Mab11	mIgG2a	BV605	Directly conjugated	Biolegend (San Diego, CA, USA)	-
IFNγ	P2G10	mIgG1	PE	Directly conjugated	BD Biosciences	-
***Anti-CPAF T cell response: Proliferation and differentiation***						
CD3	PPT3	mIgG1	FITC	Directly conjugated	Southern Biotech	-
CD4	74-12-4	mIgG2b	PE	Secondary antibody	In house	Southern Biotech
CD8α	76-2-11	mIgG2a	BV605	Biotin-Streptavidin	Thermo Fisher	Biolegend
TCRγδ	PGBL22A	mIgG1	AF647 *	Directly conjugated	Kingfisher	Thermo Fisher
CCR7	3D12	rIgG2a	BB700	Directly conjugated	BD Biosciences	-
Proliferation	-	-	Cell Trace Violet	-	Thermo Fisher	-
Live/Dead	-	-	eFlour780	-	Invitrogen	-

* Directly labeled with Alexa Fluor^TM^ 647 Antibody Labeling Kit (Thermo Fisher).

## Data Availability

The original contributions presented in this study are included in the article/Supplementary Materials. Further inquiries can be directed to the corresponding author.

## Supplementary Materials

The following supporting information can be downloaded at: https://www.mdpi.com/article/10.3390/vaccines13050468/s1, Figure S1: Schematic representation of the gating strategy for the analysis of IFN-γ production by T cell subsets; Figure S2: Schematic representation of the gating strategy for the analysis T cell proliferation; Figure S3: TNF-α production within T cell subsets in response to in vitro CPAF restimulation; Figure S4: Proliferation of γδ T cells and non-background corrected proliferation of T cell subsets; Figure S5: Local anti-CPAF IgG/IgA response pre-challenge; Figure S6: Local IgG/IgA response post-challenge.
