# Supplementary material for: A Cyclic-di-AMP Adjuvanted CPAF Protein Vaccine Is Immunogenic in Swine, but It Fails to Reduce Genital Chlamydia trachomatis Burden
Source: Vaccines (Basel). 2025 Apr 27;13(5):468. doi: 10.3390/vaccines13050468 (PMC12115861; doi:10.3390/vaccines13050468)
Supplement: Supplementary file 1 [file vaccines-13-00468-s001.zip › vaccines-3592307-supplementary.pdf]

Supplementary Material

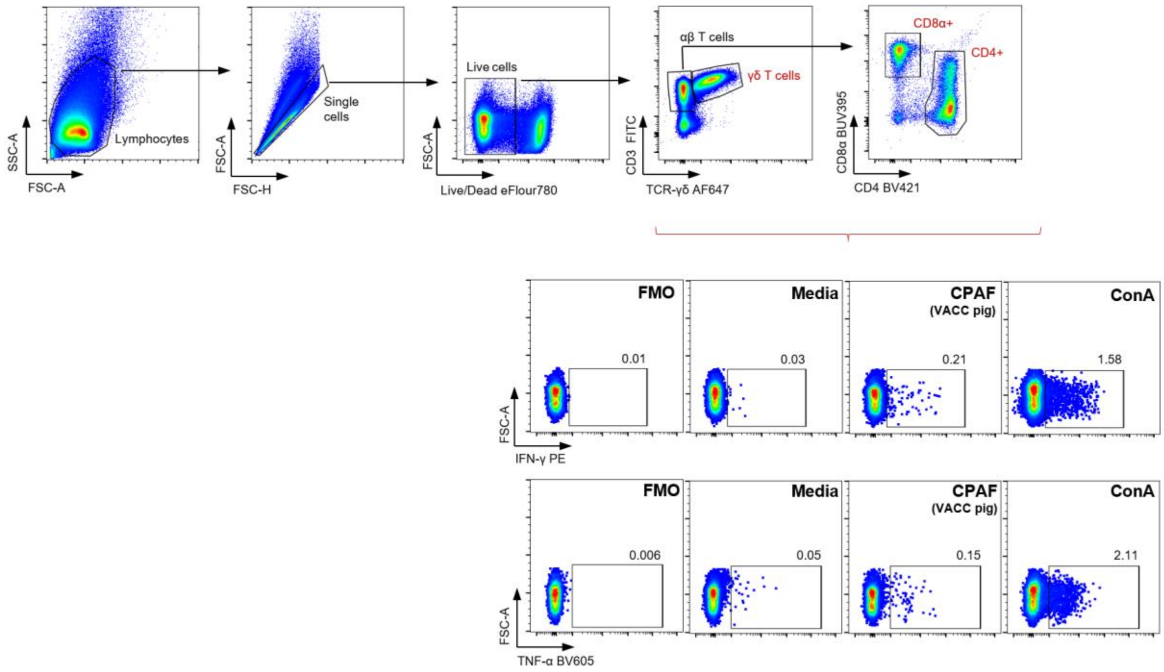

Supplementary Figure S1. Schematic representation of the gating strategy for the analysis of IFN-γ production by T cell subsets. A gate was established around lymphocytes, followed by a gate for singlets before drawing a gate surrounding intact cells. After this doublet and dead cell exclusion, γδ T cells were gated based on their TCR-γδ and αβ T cells defined as CD3+TCR-γδ-. Alpha-beta T cells were further divided into CD4 and CD8 T cells. All T cell subsets (γδ T cells, CD4 and CD8 T cells) were then analyzed for their intracellular IFNγ production as shown above under different conditions (media= negative control, CPAF= vaccine antigen, ConA= positive control). FMO = Fluorescence minus one control.

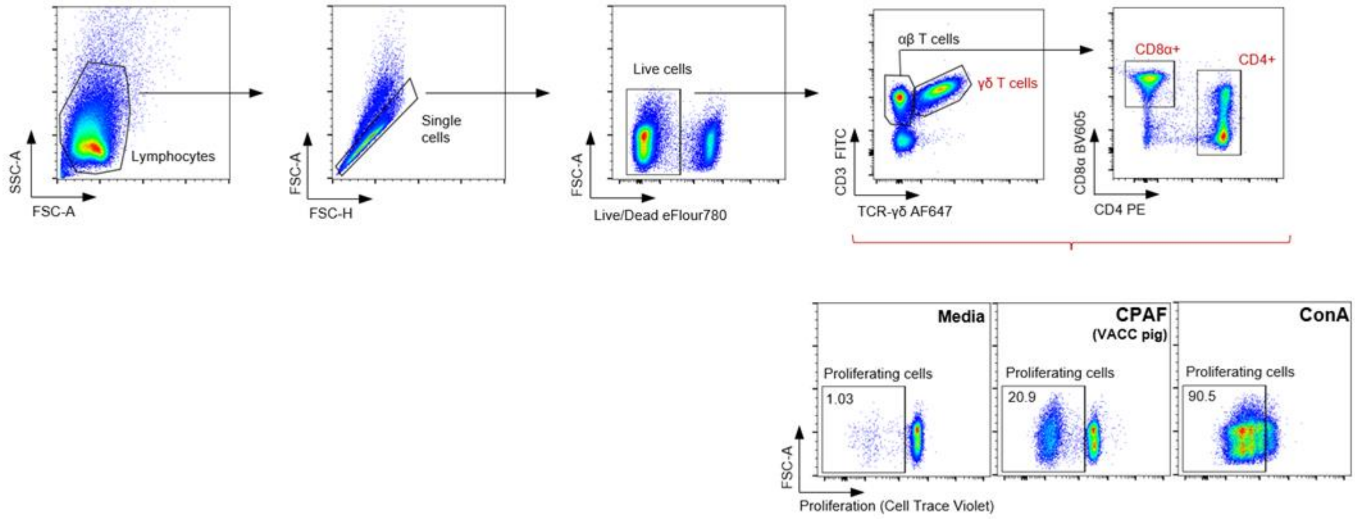

Supplementary Figure S2. Schematic representation of the gating strategy for the analysis T cell proliferation. Thawed PBMCs were rested, CellTrace Violet stained and then cultured in media (negative control), CPAF or ConA (positive control) for four days. Thereafter,

the gating strategy shown above was used to analyze T cell proliferation. A gate was established around lymphocytes, then singlets before drawing a gate surrounding living cells. After this doublet and dead cell exclusion, αβ and γδ T cells were identified based on their CD3 and TCR-γδ expression. Alpha-beta T cells were further divided into CD4 and CD8 T cells before analyzing the % of proliferating cells as shown above.

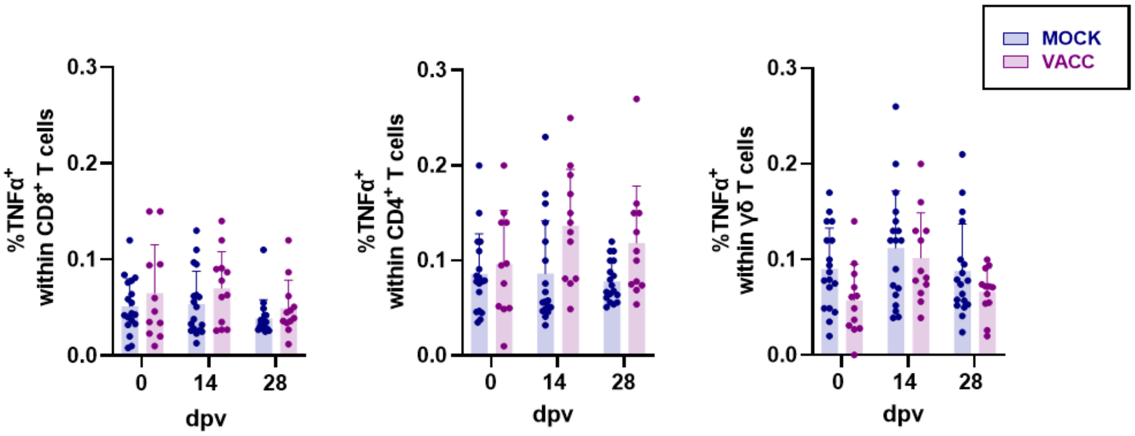

Supplementary Figure S3. TNF-α production within T cell subsets in response to *in vitro* CPAF restimulation. Previously cryopreserved PBMCs were thawed, rested and restimulated *in vitro* with CPAF. During data analysis, dead cells and doublets were excluded, as shown in **Supplementary Figure S1.** After identification of T cell subsets, their TNF-α production was analyzed. The scatter diagrams show the percentage of TNF-α positive cells with CD4, CD8 of γδ T cells at different time points. Each symbol represents data from one individual pig (n=18 for Mock and n=12 for Vaccinated). The statistical analysis was performed via GraphPad using 2-way ANOVA and Tukey multiple comparisons test. dpv= days after first vaccination.

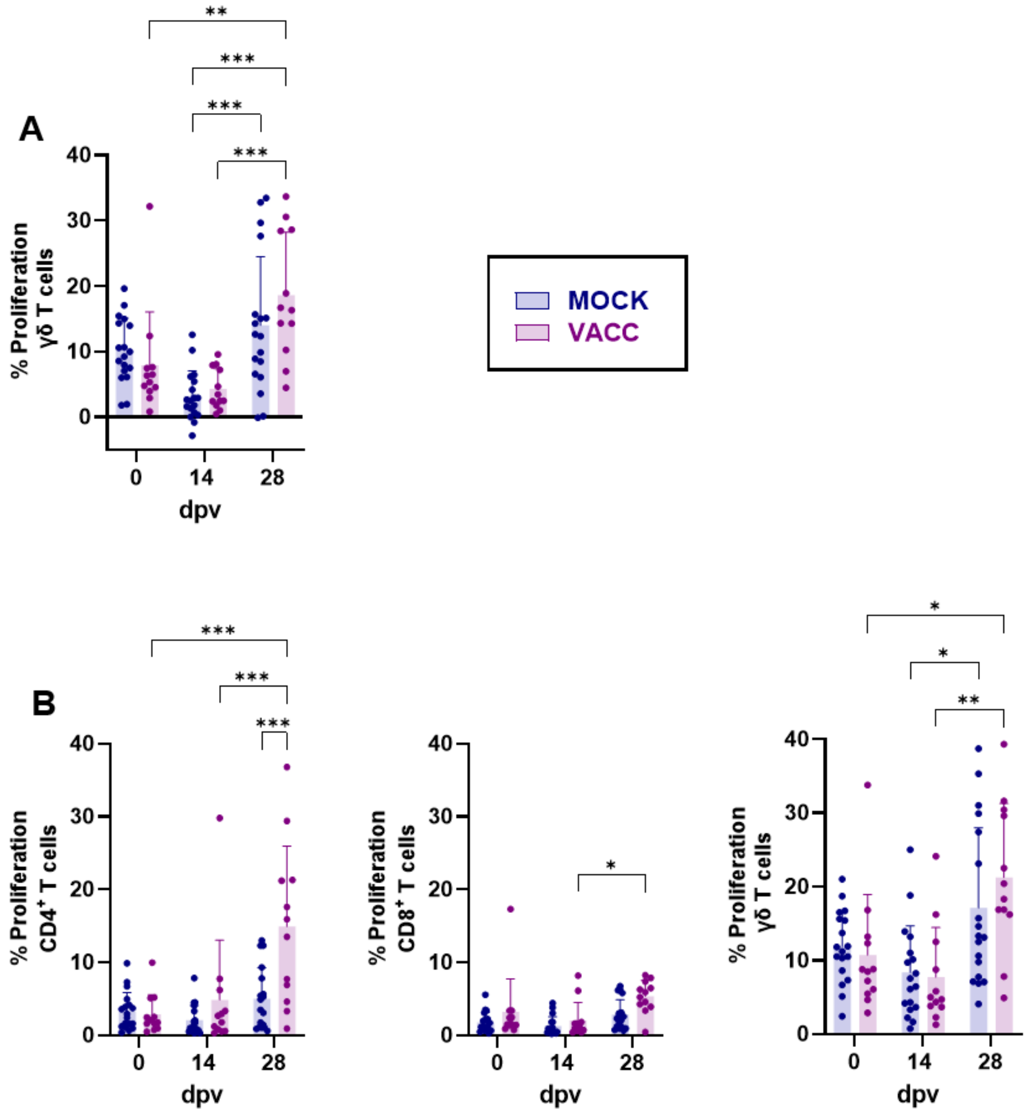

Supplementary Figure S4. Proliferation of γδ T cells and non-background corrected proliferation of T cell subsets. Previously cryopreserved PBMCs were thawed, rested and stained with CellTrace^{TM} Violet before being cultured with CPAF for 4 days. Cells were then harvested and stained as indicated in Table 1. During data analysis, dead cells and doublets were excluded, as shown in Supplementary Figure 2. (A) shows the proliferative response of γδ T cells according to their vaccination groups over time. Shown data was background corrected by subtracting the %proliferation in media (negative control, background) from the %proliferation under CPAF stimulation. (B) shows the proliferative response of all T cell subsets (CD4, CD8 and γδ T cells) without background correction. Each symbol represents data from one individual pig (n=18 for Mock and n=12 for Vaccinated). The statistical analysis was performed via GraphPad using 2-way ANOVA and Tukey multiple comparisons test. * $p < 0.05$, ** $p < 0.01$, *** $p < 0.001$.

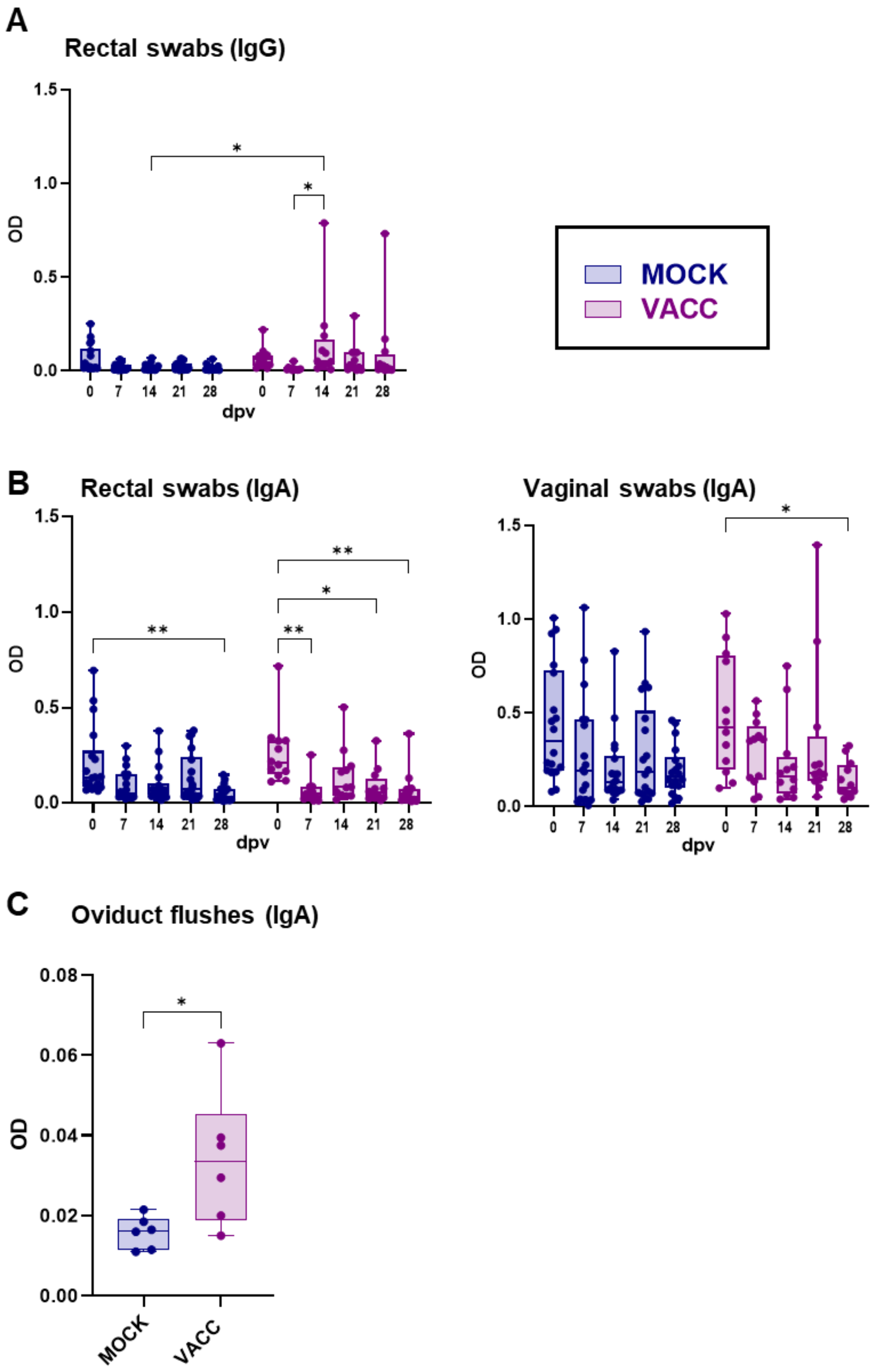

Supplementary Figure S5. Local anti-CPAF IgG/IgA response pre-challenge. (A) Immunoglobulin G (IgG) levels were quantified by anti-CPAF IgG ELISA in rectal swabs from MOCK and VACC animals. Data show optical density (OD) values. (B) IgA levels were quantified by anti-CPAF IgA ELISA in rectal and vaginal swabs. IgA levels were also quantified in oviduct flushes from pigs euthanized at day 28 (C). Each symbol represents data from one individual pig (n=18 for Mock and n=12 for Vaccinated; n=6 Mock and n=6 Vaccinated in (C)). The statistical analysis was performed via GraphPad using 2-way ANOVA and Tukey multiple comparisons test (A,B) or Mann-Whitney U test (C). * $p < 0.05$; ** $p < 0.01$. dpv= days after first vaccination.

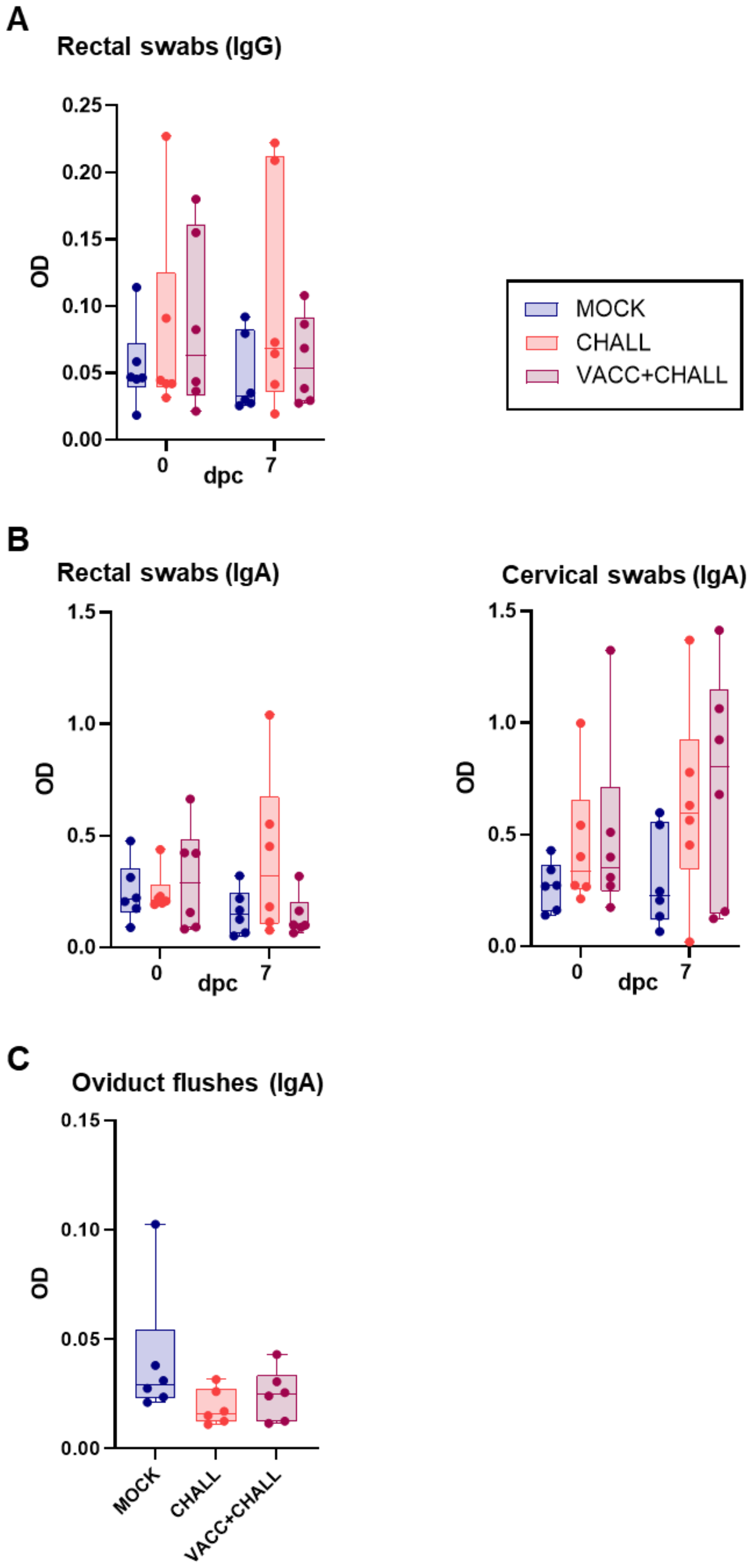

Supplementary Figure S6. Local IgG/IgA response post-challenge. Pigs were challenge with C.trachomatis 6 weeks after the first vaccination (42 dpv). Pre- and 7 days post challenge (dpc) the local IgG/IgA response was measured. IgG levels were quantified by anti-CPAF ELISA in rectal swabs (A). (IgA levels were quantified in rectal/cervical swabs (B) and oviduct flushes (C). Each symbol represents data from one individual pig (n=6 per group). The statistical analysis was performed via GraphPad using 2-way ANOVA and Tukey multiple comparisons test or one way ANOVA with Tukey multiple comparisons test (C, oviduct flushes).
